# Footprint of the COVID-19 Pandemic in India: A Study of Immune Landscape and Other Factors Shielding Mortality

**DOI:** 10.1155/2020/6692739

**Published:** 2020-12-24

**Authors:** Noura Al-Dayan, Divya Venugopal, Sugapriya Dhanasekaran

**Affiliations:** ^1^Department of Medical Lab Sciences, College of Applied Medical Sciences, Prince Sattam bin Abdulaziz University, Al Kharj, Saudi Arabia; ^2^Department of Medical Lab Sciences, College of Applied Medical Sciences, Prince Sattam bin Abdulaziz University, Wadi Ad Dawasir Campus, Saudi Arabia

## Abstract

The impact of the SARS-CoV-2 pandemic has significantly affected global health and created a world crisis. The exponentially increasing numbers of infection and mortality have made preventive measures challenging. India being a highly populated nation has so far effectively counteracted the pandemic outbreak with a significantly lower rate of mortality despite the high infection rates. The genetic architecture of the immune response genes in the Indian population, BCG vaccination, the predominantly young age group of people, and their traditional food habits might contribute to the lower rate of mortality. Human leukocyte antigens (HLA) play a vital role in triggering T cells, and natural killer (NK) cells can immediately react to eliminate infected cells. Activation of virus-specific CD4^+^ T cells and CD8^+^ cytotoxic T cells selectively targets the infected cells and strengthens the immunoregulatory system. The checkpoint for NK cell function is the engagement of killer Ig-like receptors (KIR) molecules with their respective HLA ligands overexpressed or expressed on the compromised virus-infected cells which have shown polymorphism among different ethnic groups. Here, we explore if certain KIR-HLA motifs grant Indians a survival advantage in terms of the low rate of mortality. Additionally, enhanced immunity through BCG vaccination may favor fruitful eradication of SARS-CoV-2 and provide the way out as in therapeutic intervention and vaccination strategies.

## 1. Introduction

Coronaviruses are a large family of RNA containing viruses that usually cause respiratory disease in humans [1]. Three new coronaviruses have emerged in the last two decades, spread from animals to humans causing serious and widespread disease and death. Coronaviruses are found mostly among animals like bats, camels, pigs, and cats. These viruses can sometimes be identified (spillover) in humans and can cause disease. Four (229E, NL63, OC43, and HKU1) of the seven documented coronaviruses cause moderate to mild disease, whereas three (SARS-CoV, MERS-CoV, and SARS-CoV-2) of them causes severe illness and fatality in humans. Severe acute respiratory syndrome (SARS) caused by SARS coronavirus (SARS-CoV) emerged in 2002 and disappeared by 2004. MERS coronavirus (MERS-CoV) transmitted from camels caused localized outbreaks of Middle East respiratory syndrome (MERS) in September 2012 [2]. SARS-CoV-2 is the third newly emerging coronavirus of this century that originated in December 2019 from China and was reported by the World Health Organization (WHO) as a global pandemic on 11 March 2020 [3, 4].

The host of the zoonotic virus SARS-CoV-2 includes avians that has recently passed on to humans through an intermediate host, becoming the seventh in its family that infects humans with a high mortality rate than SARS and MERS coronavirus diseases [5]. The whole-genome sequence of SARS-CoV-2 closely resembles (88% identity) bat-SL-CoVZXC21 and bat-SL-CoVVZC45 sequences which have 50% and 79% similarities with MERS-CoV and SARS-CoV, respectively, indicating it as a novel coronavirus. Notably, the early study on the novel SARS-CoV-2 virus revealed 99.98% sequence similarity among infected eight patients with four mutations being the largest difference [6]. Thus, it is similar more to both of the bat sequences and less to coronaviruses infecting humans.

The killer Ig-like receptor (KIR) family consists of several receptor molecules with different specificity in human leukocyte antigen (HLA) (human class I MHC) molecules for allelic polymorphisms. KIR expression in the human population is highly diverse and is distinctive in each natural killer (NK) cell within an individual [7]. Remarkably, it was observed in women that the KIR phenotype that is either underexpressed or overexpressed correlates with the health of the individual [7]. Additionally, KIR regulation is dependent on HLA expression and the KIR phenotype of every individual is dependent on the interactions between the receptor and the ligand. In comparison to the other KIRs, KIR2DL4 triggers a strong IFN-*γ* production by binding its ligand without any cytotoxicity [8]. Interferons are the cytokine molecules responsible for various cell signaling cascades including cell growth, cell differentiation, apoptosis, and immunoregulatory function. IFN-*γ* possesses exclusive immunoregulatory functions that are particularly significant in the innate immunity to microbial infections; it also acts as a protective shield against viral infection, particularly long-term control of viral diseases [9].

Among the Indian population, certain genetic variants of the HLA class I haplotypic groups have shown great genetic diversity associated with various diseases. A deeper understanding of the HLA genetic variations and the IFN-*γ* immunoregulatory function correlation on SARS-CoV-2 will ultimately help to develop stronger and likely more specific treatment strategies [10]. It has been explored that there is less incidence and mortality of COVID-19 in India as compared to western counterparts. This review will focus primarily on factors that may provide a shield of mortality against the COVID-19 pandemic such as the importance of HLA, modulation by KIR on INF expression, expression of angiotensin-converting enzyme 2 (*ACE2*), status of BCG vaccination, immune response of the host, and environmental and lifestyle factors.

## 2. Natural Killer Cells (NK Cells)

The assassins of defense against virus-infected or tumor-transformed cells are NK cells of innate immunity and CD8^+^ cytotoxic T cells (CTLs) of adaptive immunity. Natural killer (NK) cells are the predominant innate lymphocyte subsets divided into cytotoxic, regulatory, and tolerant NK cells which mediate antiviral reactions and thus have promising clinical use. NK cells circulate in multiple tissues throughout the body that can be formed by tissue-specific microenvironment through different combinations of cytokines [11]. NK cells are educated during development, undergo clonal expansion during infection, possess antigen-specific receptors, and generate long-lived memory cells [11]. However, the cell surface receptors that filter the unhealthy cellular targets from the healthy host cells are different for NK cells and CTLs [12]. CTLs are triggered through a T cell receptor flagged with a specific foreign peptide-loaded HLA class I molecule borne by infected cells, whereas NK cells can be activated through different receptors with inhibitory or activating function, independently or in combination, depending on the ligands presented by the target cell in a given event [13].

**Figure 1:**
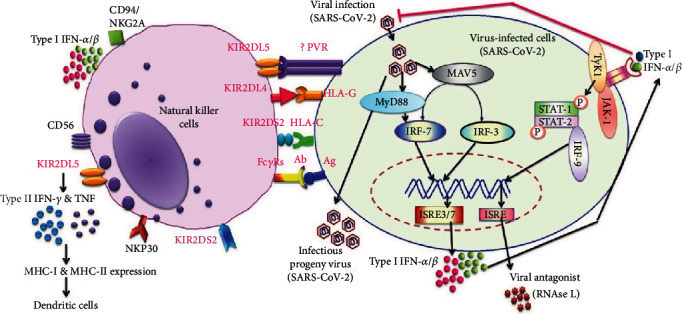
Schematic for KIR-HLA haplotype interaction and NK cell response in SARS-CoV-2 infection.

**Figure 2:**
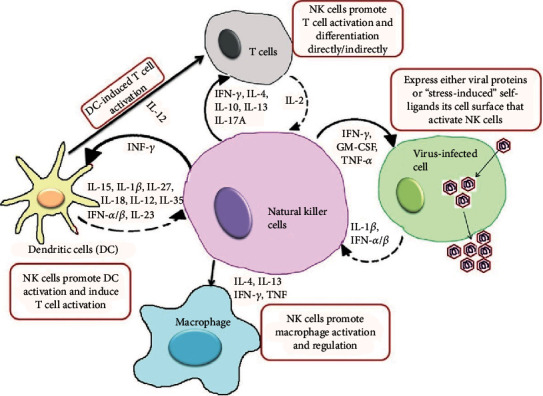
Natural killer (NK) cell action against viral infection. NK cells perform diverse functions such as innate immunity and produce soluble factors that have the ability to mediate killing target cells by their immunological functions.

Primary NK cells essentially need cytokines to facilitate their development and function, such as IL-2, IL-12, IL-15, and IL-21. Cytokines IL-15 and IL-12/15/18 will further improve NK cell cytotoxicity to maximize its efficacy for adoptive immunotherapy. NK cells utilize inhibitory receptors (KIR and Ly49) to mature and recognize self from nonself. These cells are significant producers of interferon-*γ* (cytokines) that destroy the virus-infected cells. The interaction between self-specific MHC-I receptors from KIRs (in humans) and self-MHC-I molecules plays a major role in the effective functioning of NK cells termed as “NK cell licensing” [14, 15]. However, a significant proportion of NK cells in humans lack self-specific MHC-I receptors known as “unlicensed NK cells” and display boosted functionality after preactivation with cytokines such as IL-12, IL-15, and IL-18 [16]. A recent study reveals that in humans, CD56^bright^KIR^−^ (KIR2DL4) and CD56^dim^KIR^−^ (KIR2DL5) natural killer cells can attain KIR expression upon IL-15 stimulation in the presence of stromal cells [17] (Figure 1). Activation with IL-2, IL-12, and IL-15 cytokines can enhance the *de novo* expression of KIRs and/or CD94/NK group 2 member A (NKG2A) on KIR− NKG2A− natural killer cells without feeder cells [18]. These data suggest that cytokine activation can trigger unlicensed NK cells to be stronger reactions against target cells. Functional benefits induced by IL-15 depend on the activated mTOR-regulated signal [19]; thereby, maintenance of NK cell processes implies a supportive implementation of cytokines (IL-15) in adoptive NK cell therapy. Additionally, it promotes mature NK cells to produce a large granular lymphocyte with increased cytokine production (IFN-*γ* and perforin) by coactivation with IL-2 or IL-15, resulting in enhanced cytotoxic effects [20] (Figure 2).

### 2.1. Diversity of Killer Ig-Like Receptors (KIRs) on NK Cells

Killer Ig-like receptors (KIRs) belong to the family of NK cell receptors that are different from other NK cell receptors owing to the abundant diversity found in individual-specific KIR gene content and the nucleotide sequence polymorphism of the KIR genes. In humans, there are 14 KIRs encoded by a cluster of genes on a stretch of 150 kb Leukocyte Receptor Complex (LRC) sequence found on chromosome 19q13.4 that triggers activation (3DS1, 2DS1-5), inhibition (3DL1-3, 2DL1-3, and 2DL5), or both (2DL4). The KIR family is unique for its allelic diversity, and over 30 different haplotypes have been demonstrated in different groups and populations [21]. Based on the number of extracellular Ig domains (2D or 3D), KIR is divided into two classes according to its long (L) (block lysis of NK cells) or short (S) (cytotoxic properties) cytoplasmic tail [22]. The specific blocking or activating effects of different KIRs rely on the small variations in the L and S cytoplasmic tails. The L-form of KIRs is important for the self-identification of MHC molecules and the prevention of autoimmune disorders. Furthermore, the inhibitory KIRs are considered to be evolved first, acting as ancestors to triggering KIRs [23]. The activating KIRs (short cytoplasmic tail) are truncated and lack an immunoreceptor tyrosine-based inhibitory motif (ITIM), unlike the inhibitory KIRs. Binding of MHC class I molecules to KIR inhibits NK cell activation by signaling through an associated immunoreceptor tyrosine-based inhibitory motif (ITIM). The lytic capacity of a NK cell is determined by striking a balance between the activating and inhibitory KIRs. Among the complete KIR family, the KIR2DL4 is unique from other activating KIRs as it possesses Arg residue instead of Lys in its extracellular domain, and although it contains a functional ITIM domain, it is present only on cytokine-producing population or CD56^bright^ of peripheral blood NK cells [24]. In addition, to activate NK-mediated cell lysis, the KIR2DL4 short-form associates with the Fc*γ*-RI*α* adapter protein and not with DAP1224 triggering a robust production of IFN-*γ* without inducing cytotoxicity [25]. The ligands for KIR provide specific motifs among the HLA class I molecules with HLA-C being the dominant one. HLA-C is the product of a highly polymorphic class of MHC genes.

### 2.2. Interferon-Gamma (IFN-*γ*) in Viral Infection

Interferon-gamma (IFN-*γ*) is a pleiotropic, dimerized soluble cytokine and is the only member of the type II class of interferons that regulates both the innate and adaptive immunity levels [26]. IFN-*γ* is a glycosylated protein of 25 kDa that is produced by NK cells and type 1 CD4^+^ and CD8^+^ T cells (immune IFN). IFN-*γ* has a clear role in host defense against viruses, including hepatitis B, herpes simplex, lymphocytic choriomeningitis virus, and mousepox [27]. Immune function is enhanced by Th1 cells while inhibiting Th2 cell growth and generating NK cytotoxic activity. IFN-*γ* induces MHC class I and class II gene expression on the surface of target cells and enhances antigen presentation by activating dendritic cells [28]. IFN-*γ* is the major activator of macrophages, primarily through the nitric oxide production, induction of superoxide, and NRAMP (natural resistance-associated macrophage protein) gene expression [29]. This function of IFN-*γ* is critical for host resistance against intracellular pathogens.

## 3. Why Is SARS-CoV-2 Apparently Smiling on Indians?

Since 25 March 2020, India has undergone a phase-by-phase national lockdown in the surge of COVID-19. The increasing rate of SARS-CoV-2 infection has caused various health care issues and a tremendous burden on the management of this pandemic. However, there are certain characteristics that have been brought to the foreground that makes it unique to the Indian situation. The scientific community needs to delve into these aspects and perhaps generate solutions that will be unique to India. The specific immune response to COVID-19 among Indians has not been worked out in detail. However, the reduced mortality of COVID-19 patients in India is a glaring example to indicate that there is something unique about the Indian immune climate for COVID-19. There are a few reports emerging that are highlighting the differences among Indians that may be providing protection against COVID-19 mortality. It may be likely environmental conditions, multiple biological factors, and public health response that are modulating the reduced mortality in India; however, further research in basic, clinical, and epidemiological areas is required for a better understanding of such variation among Indians as it pertains to COVID-19 response. We have tried to bring out these points coherently in this article. The lines of thought that may be worth pondering upon are described as follows.

### 3.1. KIR/HLA: Immune Advantage for Indians

- *HLA Diversity*. Viral infection downregulates the expression of HLA class I MHC molecules on the surface of infected cells evading cytotoxic T lymphocyte (CTL) response, making them potential targets for NK cell-mediated lysis. Studies have shown that the disease outcome can be influenced by the genetic variation in the host HLA class I. *In silico* analysis found that HLA-B∗46:01 had the fewest predicted binding peptides for COVID-19 and that individuals with this genotype may be particularly susceptible to COVID-19 as it was to SARS [30]. Another *in silico* analysis found that certain alleles (e.g., HLA-B∗4601) were associated with severe infection and the frequency of this allele related to SARS susceptibility was only 0.26% in Western India, compared to 13.5% in Wuhan, China [31].
- *KIR Diversity*. There are 2 main KIR haplotypes defined: group A haplotypes are characterized by the absence of all stimulatory receptors and the presence of 2DS4, and group B haplotypes are defined by the presence of one or more of the following genes: KIR2DS1-3, KIR2DS5, KIR3DS1, and KIR2DL5 [32]. Over 30 different KIR haplotypes have been recognized by genomic analysis [33]. Studies have shown that sequence polymorphisms in KIR genes can make HIV escape from NK cell-mediated immune response [34].
- *KIR*-*HLA Diversity*. Variable KIR and HLA gene families segregate independently presenting a scenario where individuals expressing KIRs lack HLA class I ligands and vice versa, whereby the diversity in the number and type of KIR-HLA combinations may modulate the outcome of the disease. The interaction of KIR to HLA class I ligands determines the threshold capacity of NK cells and controls the NK cell response. In support, there is evidence for the coevolution of KIR and HLA pairs [35]. Various studies have shown that in humans, a minimum of one KIR-HLA interaction is critical to the functional development of NK cells.

A study reported that homozygous KIR2DL3:HLA-C1 rendered protection against hepatitis B viral (HBV) infection while KIR2DL1:HLA-C2 made it susceptible [36]. One of the KIR2DL3 ligands (HLA-Cw∗07) does not impart any protection against chronic hepatitis C viral (HCV) infection [33], while HLA-DQB1∗0301 may predict spontaneous resolution of HCV following acute infection [37]. KIR2DL3:HLA-C1 has been shown to give protection in patients with HCV infection but without anti-HCV antibodies [38]. However, KIR2DL3:HLA-C1 does not have the same protective effect in HIV/HCV coinfected patients indicating that the HIV infection changes the defensive effect of KIRs [33]. In human immunodeficiency (HIV) infection, the protective effect of HLA-B27 and HLA-B57 has been documented [39]. KIR3DL1/S1 is a unique gene that encodes both the inhibitory (KIR3DL1) and activating (KIR3DS1) receptors [40]. KIR3DS1 together with its ligand HLA-B, Bw480I (isoleucine at position 80), showed slow progression to AIDS, while on their own, both made any effect on the progression. However, highly expressed KIR3DL1 alleles (KIR3DL1∗h) combined with HLA-B∗57 (a HLA-Bw480I allele) were effective against AIDS progression and viral replication which highlights a more protective role in comparison to the combined KIR3DS1/HLA-Bw480I. Moreover, HLAB∗27 alleles which contain the Bw480T motif showed greater protection against AIDS progression in the presence of KIR3DL1∗l (underexpressed alleles), suggesting that B∗27 alleles might have a greater affinity for one or more of the KIR3DL1∗l allotypes [40]. There are studies that point out certain signature motifs of KIR-HLA specific to the racial/ethnic groups. In a study of 759 unrelated individuals, most had the four well-defined inhibitory KIRs (3DL1, 3DL2, 2DL1, and 2DL2/3) but only a subset expressing all relevant HLA class I ligands was found such as HLA-Bw4 (3DL1), HLA-A3/11 (3DL2), HLA-C2 (2DL1), and HLA-C1 (2DL2/3) [41]. Two or three of these inhibitory KIR-HLA combinations are carried by a majority of Caucasians, Hispanics, and African Americans. Interestingly, one out of five individuals in these populations carries only a single receptor-ligand pair, KIR2DL3+HLA-C1. In another study, more polymorphism was observed among African Americans in the KIR2DL5 alleles [41]. This is an inhibitory orphan receptor for which no ligand has been identified. The KIR2DL5 (designated CD158f, 2DL5, and inhibitory KIR) receptor belongs to the KIR family of 60 kDa type I transmembrane glycoprotein [42] expressed on CD56^dim^ T cell subsets and NK cells, thereby regulating innate immunity [42] (Figure 1). KIR2DL5 consists of two ITIM domains in its long tail; however, one ITIM domain may cause KIR2DL5 to be less inhibitory KIRs. It forms a subfamily by connecting with KIR2DL4. The KIR2DL5 gene has two copies that are almost identical, KIR2DL5A and KIR2DL5B [43]. Nearly 57% of the African Americans and Asians carry similar frequencies of the KIR2DL5 gene; however, African Americans carry 2DL5B whereas Asians had 2DL5A [41]. Although the role of KIR2DL5 in immunity is poorly understood, it has been implicated with impaired responses to antiviral therapies, increased susceptibility to viral infection, and faster progression in Alzheimer's patients [44]. Even though it inhibits cytotoxicity in NK92 cells, the mechanism of this receptor is unclear due to the lack of a ligand. Recently, a potential ligand, the poliovirus receptor (PVR), was shown to engage KIR2DL5 protein and inhibit cytotoxicity [44]. Does the KIR2DL5 genotype bear any significance for the Indian population?

Studies have indicated the predominance of activating KIRs and group B haplotypes among the Indian population indicating that the early human migration originated from Africa through the Southern India route [45]. About 52 different types of haplotypes were observed among the Indian population, where simple 7 KIR genes and complex 15 KIR genes were reported in Indians. Based on the early report, 59% of Indians exhibit all types of inhibitory KIRs and not more than one inhibitory KIR is missing from any individual [46]. It is also reported that classical HLA class I molecule-identifying inhibitory KIRs (2DL1, 2DL3, 3DL1, and 3DL2) are observed in 83% of the Indian individuals. The most common HLA haplotype (HLA-A2-B50-DR3) is predominantly present in only the Indian population and not in the rest of the world [47]. Furthermore, the prevalence of subtype A∗0211 of the HLA-A2 allele has been reported to be distinct only in the Indian population [48]. Do Indians have a specific immune advantage over other ethnic groups by virtue of specific KIR-HLA interaction? This is interesting in the light of a study that found higher cell numbers of dendritic cells, patrolling monocytes, NK cells, CD4^+^ T cells, and naïve B cells among Indian newborns when compared to American newborns where plasmacytoid dendritic cells, CD8^+^ T cells, and total T cells were higher [49]. However, these findings need to be correlated in Indian adults. So do these signature KIR-HLA motifs make it resistant or susceptible to infection? This could partially explain the lower mortality rate found among Indian COVID-19 patients when compared with mortality rates seen in France, Italy, or the United Kingdom (Table 1).

### 3.2. Do These Probable Factors Contribute to Lower COVID-19 Mortality among Indians?

The lower mortality rates seen among Indian COVID-19 patients when compared with others could be due to certain attributes unique to Indians. This is worth considering especially when India has a higher population density (464 persons/km^2^) when compared to other countries with lower densities [50] (Table 1). The probable factors are described as follows:

- *Virulence of the Infectious Agent*. A Chinese group reported two types of SARS-CoV-2 strains identified as the original S type and mutant L type with the mutant L type being more aggressive than the original S type. A preliminary study that is yet to be peer-reviewed used integrated sequence-based analysis of SARS-CoV-2 genome samples from India, Nepal, China, Italy, and the USA and found 99% similarity with the Wuhan SARS-CoV-2 genome [51]. The novel mutation occurred in the region of spike surface glycoprotein in the Indian SARS-CoV-2 strains that was absent in Italy, the USA, and Nepal and SARS-CoV genomes. The S protein of the virion binds to the ACE2 (angiotensin-converting enzyme 2) receptor of the host cell to gain entry.
- *Angiotensin*-*Converting Enzyme 2* (*ACE2*) *Expression*. Angiotensin-converting enzyme 2 (ACE2) is the receptor for SARS-CoV-2. *In silico* analysis of South Asians revealed close proximity to East Eurasians that involves two unique polymorphisms (rs4646120 and rs2285666) in *ACE2*. Therefore, the host susceptibility of South Asians to SARS-CoV-2 will be more similar to East Eurasians [51]. miRNA are small noncoding RNA that modulate RNA silencing and gene expression. hsa-miR-27b, a unique miRNA, targets mutation found in the SARS-CoV-2 genome isolates from India [52]. This miRNA was found to inhibit ACE expression that indirectly increases ACE2 expression [51]. Replication of HIV-1 was decreased in cells overexpressing hsa-miR-27b [53]. Nine host miRNA were identified that potentially target SARS-CoV-2, of which hsa-miR-27b is the unique miRNA that can target the Indian viral genome [52]. It has been shown that SARS-CoV and COVID-19 have similar pathologies that involve a downregulation of ACE2 leading to similar respiratory distress syndrome with similar gender bias and case fatality rates [54]. It was hypothesized that certain hormonal and genetic factors could account for ACE2 overexpression that leads to a better outcome and lower death rate in females than in males. Estrogen has a protective role in SARS by directly inhibiting SARS-CoV replication [55] and upregulating ACE2 expression [56]. Furthermore, ACE2 is located on the X chromosome at sites that escape X chromosome inactivation (XCI), a mechanism that silences transcription in one of the X chromosomes of the female mammalian cells to balance the expression dosage between XX females and XY males. This silencing is not complete as nearly 10% of the genes escape inactivation and overexpress genes at the XCI sites such as ACE2 [57]. A study based on single-cell RNA-seq analysis indicated that the Asian donor had a much higher ACE2 expression cell ratio than White and African American donors [58]. Meanwhile, another study of ACE2 expression analysis using RNA-seq and microarray datasets from control lung tissues showed there were no significant differences between Asians and Caucasians or males and females [59]. Since the ACE2-expressing cells form a very small part of cells in lung tissues, conclusions can be affected by the sample size and the purity of ACE2-positive cells in the selected samples [58].
- *A Disintegrin and Metalloproteinase 17* (*ADAM 17*) *Activity*. A Disintegrin and Metalloproteinase 17 (ADAM 17) is a protein involved in inflammation and immunity (TNF-*α*, ICAM-1, and ACE2). Polymorphisms in ADAM 10, a related protein of ADAM 17, were found among Indian asthmatic patients [60]. So, polymorphisms in ADAM 17 may be likely to have an impact on the COVID-19 immune response.
- *BCG Vaccination*. Few reports have indicated that BCG vaccination may impart immunoprotective effects towards COVID-19 mortality. Countries with no mandatory program for BCG vaccination have higher COVID-19 fatality (the USA, the Netherlands, and Italy) than countries with a universal policy of vaccination (Japan) [61]. However, China despite having a program faced high mortality, due to the 10-year gap between 1966 and 1976 when the program was disbanded, thereby creating a pool of potential hosts prone/susceptible to COVID-19. Consequently, India too has a national program of BCG vaccination and it can be suggested that the protective effects have been observed in the present pandemic. The immunity imparted by BCG vaccination has been shown to extend and enhance the immune response to other nonrelated pathogens [62]. BCG induces changes in the immune system that alleviate the immune response to infections at the innate and adaptive immunity levels [62]. In innate immune cells, BCG induces histone modifications and mutations at the promotor sites of genes encoding inflammatory cytokines such as interleukin- (IL-) 1, IL-6, and tumor necrosis factor (TNF). This process is termed as “trained immunity” [63]. BCG vaccination significantly increases the secretion of proinflammatory cytokines, specifically IL-1*β*, which has been shown to play a vital role in antiviral immunity [64]. This altered trained innate immune defense system has been shown to reduce parasitemia in malarial infection [65] and protects against experimental yellow fever [66]. A study in South Africa showed a 73% reduction in respiratory tract infections in BCG-vaccinated individuals as compared to nonvaccinated individuals [67]. A similar report has been seen in multiple doses of BCG vaccination among elderly persons in Indonesia [68]. Furthermore, a reduced risk of pneumonia was also observed in BCG vaccination among elderly people in Japan [69]. In countries with universal BCG vaccination policy, reduced disease burden and mortality was observed in the initial weeks of the COVID-19 pandemic, suggesting that induced innate immunity provided by the vaccination confers a strong resistance against COVID-19 [61, 70]. Also, reports indicate that ethno-specific protection to the South Asians may be offered by BCG vaccination. Since these studies are mostly observational, definitive evidence for the causal relationship between BCG vaccination and COVID-19 fatality needs to be established.
- *Host Factors*. In diseases, the host factor determines the severity and infectivity and finally the mortality rate. The immune response of the host plays a significant part in the outcome of the disease. According to WHO, SARS-CoV-2 infection has a higher mortality rate among senior citizens of Italy (14%) [64] (above 60 years), whereas there was insignificant mortality among young people [61]. Data has shown that SARS-CoV-2 infection in countries with a high median age (Italy 45 years; Spain 47 years) is very much higher than that in countries with a lower median age (India 28 years; African continent 18 years) (WHO, https://www.who.int/) [70, 71].
- *COVID*-*19 Challenges in Rural Health Care*. The low mortality among the Indian population could also be contributed by a 65-68% population living in rural areas [72]. Despite our past victories in overcoming tuberculosis and smallpox and the absence of a vaccine, and in the wake of its high rate of infectivity, rural India is the biggest challenge for tackling COVID-19. The Indian rural health care system, which is a three-tier system composed of subcenters, primary health centers (PHC), and community health centers (CHC), has presented a shortfall: 18% at the subcenter level, 22% at the PHC level, and 30% at the CHC level (as of March 2018) [73]. Lack of infrastructure, health care workers, and quality of care has contributed to handling the pandemic in rural areas. For example, rural India has 3.2 government hospital beds per 10,000 people, and in some states, it is lower [74]. Apart from that, the absence of diligent surveillance, poor disease management, and the reverse migration of the migrant workers from urban to rural areas that started during the lockdown period have complicated and left a deep void to grasp the true statistics of the pandemic.
- *Environmental and Lifestyle Factors*. Reports have indicated that high temperatures and humidity can retard SARS-CoV-2 progression implying that Indian climatic conditions may not be conducive for virus transmission. However, recent weather reports in India have predicted otherwise in the coming future months with increased vulnerability to SARS-CoV-2. There is ample evidence to show that the spices used in Indian daily traditional food such as turmeric, cumin, garlic, pepper, ginger, cinnamon, cardamom, cloves, and fenugreek have bioactive phytochemicals that possess antioxidant, antiproliferative, antihypercholesterolemic, antidiabetic, and anti-inflammatory effects on human health [75]. Among these spices, turmeric, a golden spice, contains an active polyphenol named curcumin that possesses numerous pharmacological activities. Novel animal and human studies indicate that curcumin can affect different immune cells, such as various T lymphocyte subsets, macrophages, dendritic cells, B lymphocytes, and natural killer cells, which results in decreasing severity of various diseases with immunological etiology [76]. In a study, curcumin inhibited influenza A virus (IAV) in human lung cancer cell line A549 and the severity of the disease was decreased in the mouse after IAV infection. Heme oxygenase-1 expression was triggered *in vivo* while IAV-induced injury to the lung tissue decreased. There was inhibition of local inflammatory cytokine expression following IAV infection by curcumin. Curcumin played a role in the inhibition of NF-*κ*B signaling in macrophages enhancing I*κ*B*α* and AMPK, leading to the subsequent expression of chemokines/cytokines in response to IAV infection [76]. The antioxidant effect of curcumin might improve the protective effect on SARS-CoV-2 infection [77]. Furthermore, it also explores the role of spices in balancing blood sugar as well as type 2 diabetes, cancer, cardiovascular disease, hypertension, and AIDS. Spices, as a part of the daily diet, help to adjust the lipid profile and reduce the glucose level. Many spices like cardamom help in gastrointestinal disorders as well as help balance the cholesterol level [78]. There are studies to show that spices play a part in regulating the immune response in viral infections. Since there is evidence to show CD8^+^ T cell cross-reactivity between SARS-CoV-2 and influenza A virus [79], it is possible that curcumin modulates the immune response of SARS-CoV-2 as it does with IAV. A study showed that piperine, a bioactive compound from black pepper, inhibits the interferon inhibitory domain (PDB id 3FKE) of Ebola virus and methyltransferase (PDB id 1L9K) of dengue and has more antiviral efficacy than ribavirin [80]. Kaushik et al. [81] reported that *Z*. *officinale* extract has excellent anti-Chikungunya activity and also combats drug resistance in the Vero cell line infected with Chikungunya virus. Studies have shown antiviral effects of garlic in preclinical and clinical studies. Antiviral mechanisms include the following: blocking the entry of viruses and their fusion to host cells, inhibition of viral RNA polymerase, reverse transcription, viral replication, and enhancing host immune response. The innate antiviral immune response is increased through macrophages and natural killer cells while adaptive immunity is elevated via anti-inflammatory cytokines, T cells, and B cells. These responses have been observed in randomized clinical trials for the treatment of several viral infections that include viral hepatitis, common cold, warts, and flu [82]. So, it will be a natural progression that Indian dietary spices will modulate the COVID-19 immune response.

## 4. Differential “Cytokine Storm” among Patients Determines the Treatment

Multiple mechanisms are involved in hampering the JAK-STAT signaling cascade by proteins encoded by viruses (DNA and RNA) [83]. Moreover, viruses encode products that mimic cellular components of the interferon (IFN) signaling cascade. This cellular mimicry can significantly contribute to the antagonism of the IFN signaling and ensue the impairment of an antiviral condition. For example, vIFN-Rc, a soluble IFN receptor homolog secreted by poxvirus-infected cells, binds to IFN, thereby inhibiting them from functioning via its natural receptors to induce an antiviral response [83]. The majority of the COVID-19 patients develop mild to moderate symptoms, while some develop hyperinflammation triggered by massive cytokine/chemokine production, called a cytokine storm, which can lead to fatal pneumonia and acute respiratory distress syndrome (ARDS) [84]. Patients with severe COVID-19 have shown different cytokine profiles [84, 85]. For the first time, higher levels of interleukin- (IL-) 2, IL-7, IL-10, tumor necrosis factor (TNF), granulocyte colony-stimulating factor (G-CSF), interferon-gamma-induced protein 10 (IP-10; CXCL10), MCP-1 (CCL2), and MIP-1A (CCL3) were observed in intensive care unit (ICU) patients compared to non-ICU patients [45]. Subsequently, elevated levels of other cytokines, such as IL-1*β*, IL-1ra, IL-2R, IL-6, IL-8 (CXCL8), IL-17, interferon- (IFN-) *γ*, and GM-CSF (granulocyte-macrophage colony-stimulating factor), during severe COVID-19 infections were recorded [85]. Importantly, the elevated levels of several cytokines (IL-6, IL-10, IFN-*γ*, TNF, and IP-10) have been found in severely ill (ICU) COVID-19 patients than the mild to moderate (non-ICU) group [84, 85]. The presence of the T helper 2 cytokine IL-10, which suppresses inflammation, is a prominent feature of all reports, and an imbalance and/or exhaustion of T cells may be also involved [84].

Various approaches to harness the cytokine storm are directed at globally targeting the inflammation or neutralizing a single key inflammatory mediator and are being employed. A key cytokine of interest is IL-6, and antibodies that block the IL-6 receptor (tocilizumab and sarilumab) are currently under phase 2/3 clinical trials for the potential treatment of COVID-19 [23]. Another promising immunotherapeutic candidate for the treatment of COVID-19 is IFN-*γ*, and its potential is being worked out in a clinical trial for the JAK-STAT inhibitor (ruxolitinib) [86]. The earlier SARS-CoV infection revealed the protective effects of anti-TNF therapies, as TNF acts upstream of IL-6 [38]. Inflammatory diseases are successfully treated with several TNF-blocking antibodies (e.g., adalimumab, etanercept, and golimumab) and are being urgently recommended for COVID-19 treatment [87]. There is an upregulation of IL-10 during severe SARS-CoV-2 infection, but it may be also involved in the infiltration of inflammatory cells and lung fibrosis [88]. A report suggested that blocking IL-10, alone or in combination with programmed cell death protein 1 (PD-1), may be promising for the renewal of T cells and controlling COVID-19 pathogenesis [86]. However, the caveat is the development of chronic inflammatory disorders, and so experimental studies are required to clearly elucidate the therapeutic effect of overactivation or ablation of IL-10 for severe COVID-19. Due to the differential cytokine patterns among patients with severe COVID-19, “one-size-fits-for-all” may not be the solution for an efficacious immunosuppressive program. Therefore, it is essential for the clinicians to be well equipped with a cytokine panel, at least which includes IL-6, IFN-*γ*, and TNF-*α*, to precisely identify the needs of each patient before administration of selective immunosuppressive therapy. Obviously, a combination of immunosuppressive therapy with antiviral therapies that lower the viral burden should also be taken into consideration.

## 5. Cross-Reactivity of T Cell Immune Response Offers Immune Protection

Different coronaviruses have cross-reactive neutralizing antibodies. A report shows cross-reactive antibodies to human coronavirus (HCoV-EMC) in SARS-recovered patients [89]. As SARS-CoV and SARS-CoV-2 share the same receptor (ACE2), there is cross-reactivity of antibodies. However, some neutralizing antibodies against SARS-CoV have failed to bind SARS-CoV-2 indicating that there might be differences in the receptor-binding protein (RBD) [90]. Neutralizing antibodies formed from infection with other coronaviruses may provide protection against SARS-CoV-2, but the virus may enter cells and replicate via antibody-mediated enhancement. In such cases, the virus binds with the antibodies and this complex binds to Fc receptors on the host cells enabling viral replication. It is surmised that this may lead to the severity of SARS-CoV-2 infection [91]. It will be interesting to know whether cross-reactive antibodies provide protection to Indians or there is a lack thereof. T cell-mediated immune response plays an integral part in disease progression. Reports have shown a high homology between T cell epitopes of SARS-CoV-2 and SARS-CoV [92], and memory T cells have been known to persist for 11 years post infections [93]. Thus, this may help in T cell immune response against SARS-CoV. A study showed the presence of CD4^+^ T cells reactive against the S protein in 34% of SARS-CoV-2 seronegative healthy individuals. This S protein had homology with other coronaviruses, common cold, and SARS-CoV [94]. Even though variations in the T cell response among Indians have not been studied, it is possible that these other viruses may provide cross-reactive T cell immunity to SARS-CoV-2. There is not much evidence to show cross-reactivity between coronaviruses and other respiratory viruses; however, cytotoxic T cells directed against human papillomavirus type 16 were found to be cross-reactive to human coronavirus OC43 [95]. Another report showed CD8^+^ T cell cross-reactivity between SARS-CoV-2 and influenza A virus [79]. So, it is possible that the protective effect of the adaptive immune response with other viruses extends to COVID-19 in Indians.

## 6. Conclusion

Elucidating the interactions of HIR and KLA holds the key to the therapeutic success of COVID-19 infection. KIR-HLA interaction (MHC class I and MHC class II) has a significant influence on the immunopathogenesis of viral pathogenicity and has a controlling role on NK cell function in differentiating infected cell targets from the healthy host system. Research on COVID-19 should include KIR-HLA sequence variants, including their arrangements on recognition, signaling, development, receptor-ligand expression, effector function, and impact on disease susceptibility and resistance, especially understanding how the genetic diversity of HLA and KIR haplotypes impact the disease progression of COVID-19 and aid in identifying high-risk individuals. One of the ways would be to couple HLA typing with COVID-19 testing so that an early risk assessment can be done. If such signature motifs of HLA-KIR are identified, then predictors for either immune protection or “high risk” can be deployed, and it opens up the possibilities of peptide-based immunotherapy, or future vaccination strategies can be tailored for genotypically at-risk populations. Further work to find the significance of the unique genetic mutation in the Indian SARS-CoV-2 genomes may explain if Indians do have a genetic advantage, and mechanisms on how this advantage could be conferred on others would aid in mitigating COVID-19 mortality. Also, clinical trials and experiments are underway to realize the immunoprotective effects of BCG vaccination. Finally, the impact of environmental and lifestyle attributes providing protection against COVID-19 mortality needs research. These approaches, in the future, could preempt the pandemicity of COVID-19 and aid in the effective management of many more viruses with similar potential. The Indian and African subcontinents composed of a younger population have low mortality, whereas countries of higher median age are harshly affected.

## Figures and Tables

**Table 1:** Confirmed and death cases of SARS-CoV-2 from different countries of the world. The rate of SARS-CoV-2 mortality compared with the death rate, population density, urban population, and median age group of countries. India with the highest population density (464 persons/km^2^) shows the least mortality (1.4%) with the median age group of 28 years as per data on 19 November 2020 (https://www.worldometers.info/ and https://www.who.int/).

Country	Confirmed cases (numbers)	Deaths (numbers)	Case fatality (%)	Urban population (%)	Median age	Population density (persons/km^2^)
USA	11,085,184	1,333,742	12.0	83	38	36
Brazil	5,876,464	166,014	2.82	88	33	25
Mexico	1,009,396	98,861	9.79	34	29	66
UK	1,410,736	52,745	3.73	83	40	281
Spain	1,458,591	40,769	2.29	94	45	80
Italy	1,238,072	46,464	3.75	69	47	206
France	2,000,060	45,938	2.29	82	42	119
India	8,912,907	1,30,993	1.46	35	28	464
Russia	1,991,998	34,387	1.72	74	40	9
Argentina	1,419,736	35,745	2.51	93	32	17
Peru	938,268	35,271	3.75	79	31	26
Saudi Arabia	353,918	5,692	1.60	84	32	16
South Africa	754,256	20,433	2.70	67	28	49
Iran	788,473	42,461	5.38	75.5	32	52

## Data Availability

No data were used to support this study.
